# Effect of Ultrasonic Frequencies on the Aqueous Extraction of Polyphenols, Chlorogenic Acid, and Quercetin from the Whole Fruit of Pitaya (*Hylocereus* spp.)

**DOI:** 10.3390/molecules30153253

**Published:** 2025-08-03

**Authors:** Wei-Ting Lian, Chun-Yao Yang

**Affiliations:** Department of Food Science, Fu Jen Catholic University, New Taipei City 242062, Taiwan; wiltonlian2@gmail.com

**Keywords:** ultrasonic frequency, green extraction, pitaya, polyphenols, chlorogenic acid, quercetin, antioxidant activity

## Abstract

The effect of ultrasonic frequencies of 40 kHz/300 W (U-40) and 120 kHz/300 W (U-120) on the aqueous extraction of bioactive compounds from dried whole-fruit powders (DPs) of red-peel/white-flesh (WFP) and red-peel/red-flesh (RFP) pitayas was investigated, and shaking at 120 rpm (S-120) was used for a comparison. The effects of temperature and the solid-to-liquid ratio on the extraction efficiencies of the total phenolic content (TPC) and ferric-reducing antioxidant power (FRAP) of WFP and RFP were evaluated. The impact of extraction time on the aqueous extraction of specific compounds, namely, chlorogenic acid (CGA) and quercetin, from WFP and RFP was assessed with extraction modes of U-40, U-120, and S-120. At 40 °C and a 1/20 (g DP/mL) solid-to-liquid ratio, the use of U-40 achieved higher TPC and FRAP values at 15 min than U-120 and S-120 for WFP. The use of U-40 and U-120 extracted higher amounts of free CGA and free quercetin from WFP and RFP at 15 and 60 min than S-120 but showed different extraction efficiencies for free CGA and free quercetin. This study demonstrates that different ultrasonic frequencies can be applied in the green extraction of target bioactive compounds for use in nutraceutical foods.

## 1. Introduction

Pitaya (*Hylocereus* spp.), also called dragon fruit, is widely cultivated in subtropical and tropical regions, and it is rich in phytochemicals with pharmacological value, such as in antioxidant, anti-microbial, anti-diabetic, and anti-cancer applications [1,2,3]. Pitaya can be classified into several types according to the appearance of the peel and flesh, and the main varieties commonly consumed are *Hylocereus undatus* (red peel/white flesh), *Hylocereus polyrhizus* (red peel/red flesh), and *Hylocereus megalanthus* (yellow peel/white flesh) [1]. Pitaya contains vitamins, polysaccharides, flavonoids, betalains, and polyphenols, and it can also be used as a nutraceutical [4,5,6]. However, different varieties of pitaya show different contents of health-promoting phytochemicals, among which the main phenolic compounds are flavonoids and phenolic acid derivatives, including chlorogenic acid (CGA), caffeic acid, synaptic acid, gallic acid, ellagic acid, quercetin, isoquercetin, and rutin [7,8]. 

Tang et al. (2021) identified a total of 37 phenolic compounds, with CGA, caffeic acid, ferulic acid, and *p*-coumaric acid identified in red-fleshed pitaya and CGA, caffeic acid, ferulic acid, rutin, and isoquercitrin identified in white-fleshed pitaya [5]. Attar et al. (2022) reported that the total phenolic content and antioxidant capacity in red-fleshed pitaya were higher than those in white-fleshed ones, and some bioactive compounds, such as gallic acid, myricetin, caffeic acid, quercetin, and kaempferol, were identified in fruits of both species [9]. Chlorogenic acid (CGA, 3-*O*-caffeoylquinic acid) has beneficial effects due to its anti-hypertensive, anti-diabetic, anti-neurodegenerative, and anti-lipidemic properties [10,11]. Quercetin (3,3,4,5,7-pentahydroxyflavone) shows antioxidative, anti-inflammatory, anti-proliferative, anti-carcinogenic, anti-diabetic, and anti-viral properties [12,13]. Hence, it would be valuable to extract CGA and quercetin from pitaya for use as additives in nutraceutical foods. 

Conventional extraction methods, such as Soxhlet extraction, maceration, percolation, infusion, and decoction, are generally used when extracting bioactive compounds from plants and the fruit biomass by utilizing a solvent other than water [14]. The emerging extraction technologies frequently used are ultrasound-assisted extraction, microwave-assisted extraction, and supercritical fluid extraction, among which ultrasound, as an environmentally friendly technology, shows potential for the enhanced extraction of bioactive compounds from the internal structure of plant foods. This method has the advantages of reduced extraction time, energy use, CO emissions, and solvent while enhancing extraction yield, and water or other green solvents can be effectively employed [14]. Furthermore, the profiles of the phytochemicals extracted from plant foods are dependent on the extraction methods and the properties of the solvent and target compounds, the interaction between phenolic compounds and polysaccharides, and the affinity of the components at the interface [15]. Additionally, ultrasound with different ultrasonic frequencies could be applied to extract some specific compounds from plant foods with various impacts [16,17].

The phenolic compounds in pitaya are present in the pulp, seed, and peel [8,18]. Although the part of pitaya usually consumed is the pulp, the peel is also a rich source of polyphenols beneficial for health [18]. The processing of the edible and nonedible parts of pitaya is tremendously significant in providing high-nutritional products and reducing waste generation [19]. Using the green extraction process to extract bioactive compounds directly from the whole pitaya fruit (pulp and peel) would be a favorable route for the process’s sustainability by reducing the waste peel. 

The edible white-flesh/red-flesh parts of red-peel pitaya cultivated in Taiwan contain about 85.7%/85.8% water, 12.4%/12.3% total carbohydrates, 0.9%/1.1% crude protein, and 0.4%/0.2% crude fat, including vitamins, fatty acids, amino acids, betacyanins, and other phenolic compounds [20,21]. Due to the existence of abundant carbohydrates in pitaya, the impact of ultrasonic frequency on the extraction of phytochemicals could vary among pitaya varieties, thus influencing the extraction strategy for target compounds. In addition, Zhong et al. (2022) indicated that bound polyphenols were present in pitaya peel [18]. Past publications on the extraction of phytochemicals from pitaya have generally focused on extraction from its individual parts using either conventional or emerging extraction technologies [5,8,9,18], and the green extraction of bioactive compounds from the whole fruit using water has rarely been explored. Hence, the aim of this study was to investigate the effect of ultrasound at different frequencies on the aqueous extraction of bioactive compounds from the whole fruit (peel and flesh) of red-peel/white-flesh (WFP) and red-peel/red-flesh (RFP) pitayas containing abundant carbohydrates. The effects of extraction parameters on the total phenolic content (TPC) and antioxidant activity under various extraction modes were evaluated. Using water as a green solvent for the extraction of the valuable compounds CGA and quercetin from WFP and RFP, the impact of different ultrasonic frequencies (40 kHz and 120 kHz), shaking (120 rpm), and extraction time on the extraction efficiency of free CGA and free quercetin from WFP and RFP in the presence of abundant carbohydrates was assessed.

## 2. Results and Discussion

### 2.1. Effect of Extraction Mode

The main bioactive compounds and nutritional components in an extract generally vary across varieties, cultivated conditions, and the extraction method and conditions used [7,9,16]. Table 1 shows the effects of different extraction modes, i.e., ultrasound at 40 kHz/300 W (U-40), ultrasound at 120 kHz/300 W (U-120), and shaking at 120 rpm (S-120), on the extract yields, TPC (using gallic acid equivalent (GAE)), and FRAP (using ferrous sulfate heptahydrate equivalent (FSE)) values of the dried pitaya extracts (DPEs) obtained from WFP and RFP at 40 °C, 15 min, and a 1/20 solid-to-liquid (S/L) ratio (g/mL). It was found that the extract yields of WFP (67–69%) were higher than those of RFP (61–63%) for the tested extraction modes, and the use of U-120 produced a slightly higher amount of extract for both WFP and RFP. The results revealed that more hydrophilic or water-soluble compounds could be extracted from WFP than from RFP.

As shown in Table 1, for WFP, the TPC and FRAP values obtained when using U-40 were higher than those obtained when using U-120 and S-120, with significant differences. For RFP, the TPC values followed the order of U-40 > S-120 ≥ U-120, with a significant difference between U-40 and S-120 but an insignificant difference between S-120 and U-120. 

For WFP, the FRAP values followed the order of U-40 > S-120 ≥ U-120, which was consistent with the order of the TPC values; for RFP, the order of the FRAP values almost corresponded to that of the TPC values. This suggests that the antioxidant activity is mainly contributed from the polyphenols in both types of pitaya. The results demonstrate that the use of U-40 is more beneficial for enhancing the extraction efficiency of polyphenolic compounds from WFP and RFP but has different impacts.

When applying ultrasound in the extraction system, cavitation bubbles form in the liquid medium when the negative pressure in the rarefaction phase of an acoustic wave reaches a certain threshold value, which varies according to the combination of parameters, such as the hydrostatic pressure level, liquid temperature, and change rate of local pressure [22]. Nguyen et al. (2017) reported that the threshold of the mechanical effect was nearly equal to that of the cavitation effect at an ultrasonic frequency of less than 98 kHz but was significantly greater at a high frequency [23]. Hence, when using U-40 or U-120, the ultrasonic impact on the variations in the TPC values of WFP and RFP might be attributed to whether the mechanical or cavitation effect dominated. Bu and Alheshibri (2021) also reported that the bubbles generated at a lower frequency were larger than those generated at a higher frequency, with the released energy intensity approximately inversely proportional to the ultrasonic frequency [24]. The high energy intensity and cavitation effect achieved in the water medium using U-40 better facilitate the extraction of polyphenols than those achieved using U-120 and S-120; in the latter, hydromechanical stress is generated via shaking, promoting mixing and the interaction between the compounds and solvent to different extents [25].

### 2.2. Effect of Temperature 

The effect of temperature (30 to 60 °C) on the aqueous extraction of bioactive compounds from WFP and RFP was explored using U-40 with a 1/20 (g/mL) S/L ratio and a 15 min extraction time. The extract yield, TPC, and FRAP results of the dried whole-fruit powders (DPs) are shown in Table 2. The extract yield of WFP (65.6–68.0%) was greater than that of RFP (60.7–62.0%) at various tested extraction temperatures, and higher extract yields could be obtained above 40 °C for both WFP and RFP.

The TPC and FRAP values of WFP at an extraction temperature of 40 °C were higher than those at 30 °C, and the FRAP value of WFP was 6.470 mg FSE/g DP at 40 °C, showing a significant increase with the increase in temperature from 30 °C to 40 °C; in addition, the TPC and FRAP values of RFP slightly increased with the increase in temperature, but they showed insignificant differences. Additionally, the TPC and FRAP trends in both WFP and RFP were similar, indicating that the antioxidant activity could be related to the polyphenols in both types of pitaya; this also revealed that higher temperatures increase the penetration of the solvent and the solubility of the solutes and facilitate the transport of solutes from solid material [16,26].

### 2.3. Effect of Solid-to-Liquid Ratio

The S/L ratio can affect the extraction efficiency of bioactive compounds from plant materials, as well as the extract yield, antioxidant activity, and polyphenols in the extract. In this study, various S/L (1/10 to 1/40 g/mL) ratios were employed under conditions of 40 °C and a 15 min extraction time using U-40. For WFP and RFP, the extract yield, TPC, and FRAP values of the DPs are displayed in Table 3.

As shown in Table 3, the extract yield of WFP was 73.360% at a 1/40 S/L ratio, and it was significantly higher than that at 1/10 to 1/30 (g/mL) S/L ratios; at an S/L ratio of 1/20 (g/mL), the FRAP value of WFP was 6.470 mg FSE/g DP, which was slightly higher than that at other S/L ratios, but there was no significant difference. For RFP, better extract yields were obtained, 61.963% and 64.820% at 1/20 and 1/30 (g/mL) S/L ratios, respectively, as well as higher FRAP values, 19.407 and 18.525 mg FSE/g DP at 1/30 and 1/20 (g/mL) S/L ratios, respectively.

The TPC values of RFP or WFP obtained using different S/L ratios and U-40 are shown in Table 3. For RFP, the TPC values followed the order of TPC for 1/30 (S/L) ≥ TPC for 1/20 (S/L) > TPC for 1/40 (S/L) ≥ TPC for 1/10 (S/L), showing the same trend as the FRAP values. This phenomenon suggests that, at a higher S/L ratio (with less solvent), the cavitation effect could be somewhat impeded due to the higher viscosity of the solution [27].

Moreover, it should be noted that pitaya contains a large amount of carbohydrates [20,21]. When using water as a solvent in the extraction of polyphenols, more hydrophilic carbohydrates were extracted and accounted for the majority of the extract yield, and the total polyphenol content in the extract yield seemed to be rather small in comparison. It was found that, when using U-40 with the tested S/L ratios, although the extract yields of WFP were larger than those of RFP, the TPC values of WFP were smaller than those of RFP under the same extraction conditions. A reason for this may be that, under the same extraction conditions, the cavitation effect on the microstructures of WFP and RFP had different impacts on the transport of ingredients within the internal structure and on the interaction between the polyphenols and carbohydrates [16,18,26].

### 2.4. Effect of Extraction Time

**Figure 1:**
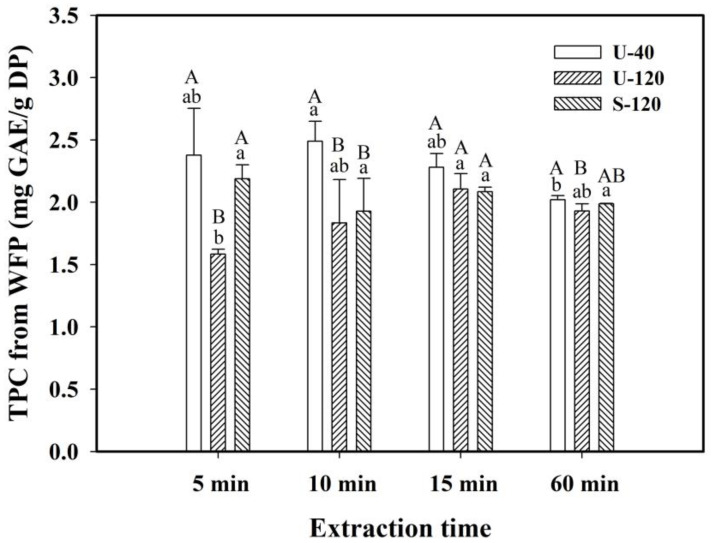
Variations in the total phenolic content (TPC) extracted from white-flesh (WFP) pitaya at different extraction times. Conditions: 40 °C, 1/20 S/L ratio (g/mL). U-40: ultrasound at 40 kHz and 300 W; U-120: ultrasound at 120 kHz and 300 W; S-120: shaking at 120 rpm. Data are expressed as mean ± standard deviation (*n* = 3). Values with different uppercase letters for the same extraction time or values with different lowercase letters for the same extraction mode were found to be significantly different (*p* < 0.05) using Duncan’s multiple range test.

The effects of extraction time (5, 10, 15, and 60 min) on the TPC and FRAP values of the DPs obtained from WFP and RFP were evaluated with different extraction modes (U-40, U-120, and S-120) at 40 °C and a 1/20 (g/mL) S/L ratio. The TPC values of WFP are shown in Figure 1.

As shown in Figure 1, when using U-40, the TPC value at 10 min (2.489 mg GAE/g DP) was significantly higher than that at 60 min (2.020 mg GAE/g DP) and was slightly higher than that at 5 and 15 min with insignificant differences, revealing that using U-40 could lead to rather stable TPC values for 5 to 15 min with a decrease until 60 min. Although the TPC value at 15 min was slightly higher when using U-40 than when using U-120 and S-120 with insignificant differences, that at 10 min was significantly higher when using U-40 than when using U-120 and S-120, thus showing that U-40 could release a larger energy intensity than U-120 and S-120. However, the TPC value at 60 min was still higher when using U-40 than when using U-120 with a significant difference. This indicates that, with a longer extraction time, the phenolic compounds slightly degraded to various extents when using U-40 and U-120.

Moreover, when using U-120, the TPC value at 15 min (2.106 mg GAE/g DP) was significantly higher than that at 5 min (1.584 mg GAE/g DP) but was only slightly higher than that at 10 and 60 min with insignificant differences; this suggests that the possible degradation of phenolic compounds could be compensated within the first 15 min of extraction due to the enhanced extraction of U-120 at a lower energy intensity. Wang et al. (2020) investigated the degradation behavior of polyphenols in a model aqueous extraction system under ultrasound and reported that the hydroxyl radical concentration increased linearly with the sonication time, leading to the degradation of caffeic acid on a time scale of 25 min; thus, free radical scavengers could minimize the degradation of polyphenols by inhibiting hydroxyl radicals [26]. This revealed that the extraction efficiency of polyphenols was influenced to various extents by the combined effects of local hot spots generated by ultrasound, cavitation caused by different ultrasonic frequencies, and the interaction between extracted compounds [18,28].

**Figure 2:**
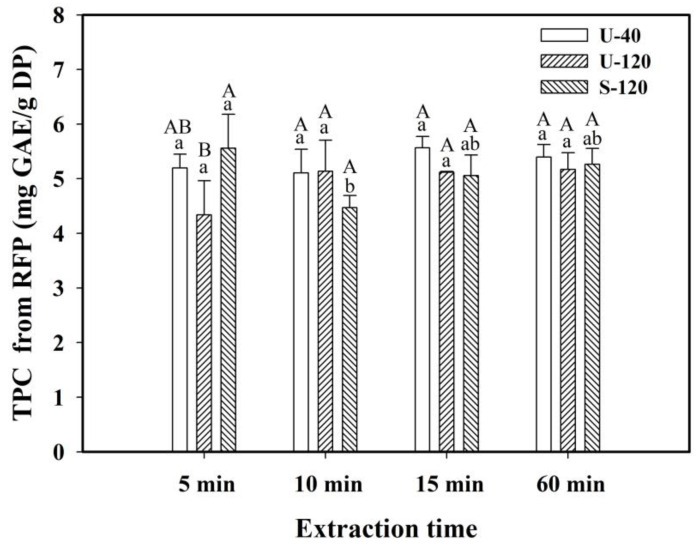
Variations in the total phenolic content (TPC) in red-flesh (RFP) pitaya at different extraction times. Conditions: 40 °C, 1/20 S/L ratio (g/mL). U-40: ultrasound at 40 kHz and 300 W; U-120: ultrasound at 120 kHz and 300 W; S-120: shaking at 120 rpm. Data are expressed as mean ± standard deviation (*n* = 3). Values with different uppercase letters for the same extraction time or values with different lowercase letters for the same extraction mode were found to be significantly different (*p* < 0.05) using Duncan’s multiple range test.

The TPC values of RFP are displayed in Figure 2. For RFP, the TPC values of the DP at 15, 60, 5, and 10 min using U-40 were 5.568, 5.396, 5.200, and 5.109 mg GAE/g DP, respectively, with insignificant differences from the 5 to 60 min extraction times. When using U-120, the TPC values at 60, 10, and 15 min were 5.170, 5.139, and 5.119 mg GAE/g DP, respectively, with insignificant differences for these extraction times. The results revealed that, for RFP, the degradation levels of polyphenols were not significant after 15 min of extraction when using either U-40 or U-120, implying that their degradation could be compensated by sonication, which enhanced the release of phenolic compounds. However, although after 15 min of extraction, the TPC values of RFP were slightly higher when using U-40 than when using U-120 and S-120 with insignificant differences, using U-40 still had the benefits of obtaining extracts from RFP with stable and higher polyphenol contents.

Table 4 displays the effect of extraction time on the FRAP values of the extracts from WFP and RFP using different extraction modes at 40 °C and a 1/20 S/L ratio (g/mL). The results show that the FRAP values of the extracts obtained from RFP were larger than those obtained from WFP for each extraction mode and time. The FRAP results of WFP and RFP could be related to the TPC values of WFP (Figure 1) and RFP (Figure 2), respectively, among which the TPC values of RFP were greater than those of WFP for the same extraction mode at each extraction time, demonstrating that polyphenols mainly contributed to the antioxidant activity of the extract. 

When using U-40, the FRAP value of WFP was slightly higher at 10 min than at 5, 15, and 60 min with insignificant differences, revealing a slight decrease after 10 min. In addition, with a 15 min extraction time, the FRAP value obtained using U-40 was larger than that obtained using U-120 and S-120 with significant differences, but, with other extraction times, it was slightly higher than that obtained using U-120 and S-120 with insignificant differences, showing that U-40 can achieve relatively higher and stable FRAP values for WFP. For RFP, the FRAP values obtained at each extraction time using U-40, U-120, and S-120 showed insignificant differences; however, they showed a slight decrease at 60 min. This suggests that, for RFP, an extraction time of 15 min is better for achieving an aqueous extract with more stable antioxidant activity.

### 2.5. Aqueous Extraction of Chlorogenic Acid and Quercetin 

Chlorogenic acid (CGA) and quercetin are useful phytochemicals beneficial for human health, and their extraction from pitaya is valuable. The effects of extraction time and mode on the aqueous extraction of free CGA and free quercetin were assessed at 40 °C and a 1/20 S/L ratio (g/mL). Pitaya contains an abundance of carbohydrates [20]; thus, when using water as a green solvent to extract the free CGA from pitaya, a large amount of carbohydrates could be simultaneously extracted and interact with CGA during extraction such that the interaction between CGA and carbohydrates at various extraction durations and modes could occur to different extents [16,17]. The free CGA and free quercetin results are shown in Table 5 and Table 6, respectively.

As displayed in Table 5, the free CGA contents in the DPs of WFP were larger than those in RFP at each extraction time and under each extraction mode. When using U-40, the free CGA content in WFP at 15 min was higher than that at 5 min with a significant difference but was slightly higher than that at 10 and 60 min with insignificant differences; furthermore, the CGA content at 15 min was found to have a larger standard deviation, which was speculated to result from the different degrees of interaction between CGA and carbohydrates during the extraction of the independent samples. Although the free CGA content extracted using U-40 slightly decreased at 60 min, the difference was insignificant.

When using S-120, the free CGA contents in WFP were significantly higher at 5 and 10 min than at 15 and 60 min. This phenomenon revealed that, after using S-120 for 15 min, severe degradation of the free CGA and possible interactions between CGA and carbohydrates occurred; this degradation was speculated to have been caused by the action of hydromechanical stress induced by shaking [29]. The results also revealed that the free CGA contents extracted at 15 and 60 min were higher when using U-40 and U-120 than when using S-120, showing that using ultrasound with a 15 min extraction time could provide a better CGA content in the extract than using shaking. Additionally, when using U-120, the free CGA content was slightly higher at 15 min than at the other extraction times but with insignificant differences, demonstrating that the free CGA in WFP was more stable throughout the extraction duration when using U-120 than when using U-40, as well as revealing that U-40 and U-120 have different impacts on the extraction of free CGA.

For RFP, free CGA was not detected at 5 min when using U-120 or S-120. However, when using S-120, the free CGA content was significantly higher at 10 min than at 60 and 15 min, indicating the severe degradation of free CGA after 15 min. Additionally, when using U-120, the free CGA content in RFP at 60 min was higher than that at 15 min with a significant difference, but it was slightly higher than that at 10 min with an insignificant difference. When using U-40, only a slight decrease was observed in the free CGA content in RFP from 10 to 60 min, implying a lower level of degradation of free CGA. These phenomena in RFP could be attributed to either the combined effects of free CGA interacting with carbohydrates and various levels of cavitation with local hot spots generated by different ultrasonic frequencies or to hydromechanical stress with fluid shear when using shaking [16,26,29].

Table 6 shows the effect of extraction time on the free quercetin content in the DPs of WFP and RFP when using different extraction modes. It was found that, under the same extraction mode and conditions, the free quercetin contents in WFP were larger than those in RFP. When using U-120, the free quercetin content in WFP was significantly higher at 60 min than at 5, 10, and 15 min, with no observable degradation of free quercetin after 15 min. Conversely, when using U-40, although having a larger standard deviation for a similar reason to the free CGA, the free quercetin content at 15 min was higher than that at 5 min with a significant difference, but it was slightly higher than that at 10 and 60 min with insignificant differences, showing possible degradation with a long extraction time (60 min) when using U-40 for WFP. This was speculated to result from the generation of free radicals in the aqueous solution and the thermal effects of the generation of local hot spots during sonication, leading to the degradation of polyphenols when using U-40 for ultrasonic irradiation for a long time (60 min) [29]. When using S-120, the significant increase in the free quercetin content from 15 to 60 min revealed a distinct effect of hydromechanical stress with fluid shear by shaking on the aqueous extraction of free quercetin from WFP.

For RFP, the free quercetin contents using both U-40 and U-120 significantly increased with an increasing time from 15 to 60 min, but with a slight variation in enhancement, showing that different ultrasonic frequencies had different impacts on the extraction efficiencies of free quercetin. In addition, at 5 and 10 min, free quercetin was not detected in RFP when using U-40 or U-120; moreover, when using S-120, no free quercetin could be detected at 5, 10, or 15 min. Quercetin is a more hydrophobic compound, with its solubility being 0.0000653 of mole fraction at 314.1 K in water [30]. Hence, using water to extract free quercetin from the internal structure of plant foods is limited not only by its solubility in water but also by the difficulty of its transport into the bulk solution, especially in the presence of abundant carbohydrates that could interact with polyphenols in pitaya. However, severe destruction of the microstructure and cell walls under ultrasonic irradiation is beneficial for enhancing the extraction efficiency of free quercetin, even when using water as a solvent. The results show that, for RFP, the highest free quercetin content was obtained with a 60 min extraction time, and the free quercetin content in RFP was significantly higher when using U-40 than when using S-120; this demonstrates that the extraction of hydrophobic free quercetin was effectively enhanced when using ultrasound due to the significant cavitation effect induced by sonication, especially when using the high-intensity and low-frequency ultrasound of U-40. The results indicate that ultrasound at 40 kHz and 120 kHz can promote the extraction efficiency of free quercetin better than shaking.

## 3. Materials and Methods

### 3.1. Materials 

The chemical reagents 2,4,6-tris(2-pyridyl)-s-triazine, gallic acid, rutin, and ferric chloride were purchased from Merck KGaA (Darmstadt, Germany). Folin–Ciocalteu phenol reagent was purchased from Sigma-Aldrich Co. (St. Louis, MO, USA). Chlorogenic acid (≥95% purity) purchased from Merck KGaA (Darmstadt, Germany) and quercetin (97% purity) purchased from Thermo Fisher Scientific (Waltham, MA, USA) were used as standards in analyses. Other chemicals were purchased from Bionovas biotechnology Co. (Toronto, ON, Canada), J.T. Baker Chemical Company (Phillipsburg, NJ, USA), Merck KGaA (Darmstadt, Germany), and Taiwan Sugar Corporation (Tainan City, Taiwan).

For the experiments, red-peel/white-flesh pitaya (WFP) was purchased in July 2023 and red-peel/red-flesh pitaya (RFP) was purchased in December 2022 from an orchard in Erlin, ChangHua County, Taiwan (R.O.C.). After cleaning the surface of the pitaya with water, the scale leaves on the skin were cut off, and the head and stalk were removed. Then, the fruit was sliced into pieces for freeze-drying. The dried materials were ground and subsequently screened using a 40-mesh sieve to obtain dried whole-fruit powders (DPs), which were preserved in a drying cabinet until use. In addition, the moisture contents in WFP and RFP were determined to be 4.17 ± 0.04 % and 4.47 ± 0.01 % (*n* = 3) according to the method of Hsu and Yang (2024) [31].

### 3.2. Ultrasound-Assisted Extraction of Bioactive Compounds

The aqueous extraction of bioactive compounds from the DPs was carried out in a constant-temperature bath system, which was equipped with ultrasound at 300 W and a 40 kHz (or 120 kHz) frequency (LEO-3002S or LEO-3002H, LEO Ultrasonic Co., New Taipei City, Taiwan). Then, 1 g of the DP and definite volumes of de-ionized water were introduced into a 250 mL flask in a selected solid-to-liquid (S/L) ratio (grams of DP per mL of water, g/mL) at the set temperature. Extraction was then performed under ultrasound (40 kHz/300 W (U-40) or 120 kHz/300 W (U-120)); extraction was also conducted without ultrasound but with shaking at 120 rpm (S-120) (reciprocal shaking bath, Model B602D, Firstek, Taipei, Taiwan) for a comparison. Upon the completion of extraction, the liquid was separated from the solid–liquid mixture via centrifugation at 6000 rpm for 15 min. Afterward, freeze-drying was used to remove the water from the liquid solution, and dried pitaya extract (DPE) was obtained. The yield of extract from the DPs was estimated using Equation (1):

Extract yield (% of DP) = (weight of DPE/weight of DP) × 100%(1)

### 3.3. Determination of Total Phenolic Content

The TPC in the DPE was determined through the method of Kujala et al. (2000) with modifications using the gallic acid equivalent (denoted as GAE) [32]. A liquid sample of the DPE was prepared in a concentration of 5 mg/mL by re-dissolving it in water. The liquid sample (250 μL) was used to react with Folin–Ciocalteu reagent (1N, 250 μL) at 25 °C for 5 min. Afterward, 500 μL of 20% Na_2_CO_3_ was introduced into the mixture and reacted for 8 min. A spectrophotometer (Hitachi, Ratio Beam Spectrophotometer U-5100, Tokyo, Japan) was used to measure the absorbance of the above mixture at 730 nm. The TPC is expressed as mg GAE/g DPE or mg GAE/g DP, obtained by multiplying mg GAE/g DPE with g DPE/g DP.

### 3.4. Determination of Ferric-Reducing Antioxidant Power

By slightly modifying the method of Madane et al. (2020), the antioxidant activity of the DPE was evaluated according to the FRAP using the ferrous sulfate heptahydrate equivalent (denoted as FSE) [33]. A reagent for analyzing the FRAP was prepared and stored at 37 °C. Then, 15 μL of the liquid sample of the DPE (5 mg/mL in water), 285 μL of FRAP reagent, and 500 μL of de-ionized water were mixed to react at 37 °C for 4 min. The absorbance of the mixture at 593 nm was measured to determine the FRAP of the DPE sample. The FRAP is expressed as mg FSE/g DPE or mg FSE/g DP.

### 3.5. HPLC Analysis of CGA and Quercetin

An analysis of the CGA and quercetin contents in the DPE was performed with high-performance liquid chromatography (HPLC) following the method of Chen and Yang (2020) with a slight modification [34]. In the HPLC system, a Mightysil RP-18 GP column (5 μm, 250 mm × 4.6 mm, Kanto Chemical Co., Tokyo, Japan) and a UV–VIS detector (Hitachi Chromaster 5420 UV-VIS detector, Hitachi, Ltd., Tokyo, Japan) at a wavelength of 350 nm were used. A liquid sample of the DPE was prepared by re-dissolving it in 20% aqueous ethanol for an HPLC analysis at 30 °C. The mobile phase was solvent A (acetonitrile/0.1% trifluoroacetic acid at 10/90 (*v*/*v*)) and solvent B (acetonitrile), and the flow rate was 1.0 mL/min. The gradients of the mobile phase were set for solvent A as follows: 100 to 88.9% at 0 to 10 min, 88.9 to 66.7% at 10 to 35 min, 66.7 to 0% at 35 to 40 min, 0% at 40 to 45 min, 0 to 100% at 45 to 46 min, and 100% at 46 to 50 min.

### 3.6. Statistical Analysis

Each experimental result was obtained using three independent samples and is expressed as mean ± standard deviation (*n* = 3). For statistical analysis, a one-way ANOVA with Duncan’s multiple range test was performed using IBM SPSS Statistics 20 (IBM Corp., Armonk, NY, USA), with significant differences set at *p* < 0.05.

## 4. Conclusions

In this study, the impact of ultrasonic frequencies on the aqueous extraction of bioactive compounds from WFP and RFP was investigated. The results demonstrated that U-40 was beneficial for enhancing the extraction efficiency of polyphenolic compounds from WFP and RFP but had different impacts. For WFP, the TPC value at 10 min was higher when using U-40 than when using U-120 and S-120 with significant differences, and it remained significantly higher at 60 min when using U-40 than when using U-120, indicating that U-40 and U-120 caused different levels of polyphenol degradation with a longer extraction time. For RFP, although the TPC values at 15 min were slightly higher when using U-40 than when using U-120 and S-120 with insignificant differences, U-40 was still beneficial for obtaining stable and higher polyphenol contents in RFP extracts. Additionally, the free CGA in WFP was more stable throughout the extraction duration when using U-120 than when using U-40, revealing that these methods have different impacts on the extraction of free CGA. When using S-120, severe degradation of the free CGA in WFP occurred after 15 min. Moreover, under the same extraction mode and conditions, the free quercetin contents in WFP were higher than those in RFP. The free quercetin content in RFP was significantly higher when using U-40 than when using S-120, showing that ultrasound effectively enhanced the extraction of hydrophobic free quercetin. The results of this study can be used as a reference when selecting the strategy for the green extraction of target bioactive compounds from whole pitaya fruit, promoting sustainability while simultaneously reducing waste generation, and green extraction could serve as potential technology to promote the value of pitaya for use in nutraceutical foods.

## Figures and Tables

**Table 1:** Effect of extraction mode on the extract yield, total phenolic content (TPC), and ferric-reducing antioxidant power (FRAP) of the dried whole-fruit powders (DPs) from white-flesh (WFP) and red-flesh (RFP) pitayas.

Type of Pitaya	Extraction Mode	Extract Yield (% of DP)	TPC (mg GAE/g DPE)	FRAP (mg FSE/g DPE)
WFP	U-40	67.103 ± 1.057 ^B^	3.399 ± 0.181 ^A^	9.646 ± 0.407 ^A^
	U-120	69.027 ± 0.952 ^A^	3.050 ± 0.148 ^B^	7.567 ± 0.320 ^B^
	S-120	67.137 ± 0.649 ^B^	3.105 ± 0.049 ^B^	8.285 ± 0.611 ^B^
RFP	U-40	61.963 ± 1.240 ^a^	8.985 ± 0.180 ^a^	29.903 ± 1.695 ^a^
	U-120	63.173 ± 1.023 ^a^	8.105 ± 0.119 ^b^	28.415 ± 0.615 ^a^
	S-120	62.173 ± 2.440 ^a^	8.125 ± 0.290 ^b^	29.339 ± 1.468 ^a^

Conditions: 40 °C, 15 min extraction time, 1/20 S/L ratio (g/mL). U-40: ultrasound at 40 kHz and 300 W; U-120: ultrasound at 120 kHz and 300 W; S-120: shaking at 120 rpm; DPE: dried pitaya extract. Data are expressed as mean ± standard deviation (*n* = 3). Values with different superscript letters in the same column were found to be significantly different (*p* < 0.05) using Duncan’s multiple range test.

**Table 2:** Effect of temperature on the extract yield, total phenolic content (TPC), and ferric-reducing antioxidant power (FRAP) of the dried whole-fruit powders (DPs) from white-flesh (WFP) or red-flesh (RFP) pitaya using U-40.

Type of Pitaya	Temperature (°C)	Extract Yield (% of DP)	TPC (mg GAE/g DP)	FRAP (mg FSE/g DP)
WFP	30	65.600 ± 0.782 ^B^	2.213 ± 0.092 ^B^	4.643 ± 0.369 ^B^
	40	67.103 ± 1.057 ^AB^	2.280 ± 0.111 ^AB^	6.470 ± 0.179 ^A^
	50	67.967 ± 0.627 ^A^	2.346 ± 0.181 ^AB^	6.349 ± 0.408 ^A^
	60	67.897 ± 1.401 ^A^	2.536 ± 0.166 ^A^	6.824 ± 0.186 ^A^
RFP	30	60.673 ± 0.943 ^a^	5.355 ± 0.768 ^a^	18.605 ± 3.034 ^a^
	40	61.963 ± 1.240 ^a^	5.568 ± 0.205 ^a^	18.525 ± 1.017 ^a^
	50	61.620 ± 1.846 ^a^	5.613 ± 0.254 ^a^	19.528 ± 0.369 ^a^
	60	61.493 ± 1.557 ^a^	5.425 ± 0.171 ^a^	19.951 ± 1.296 ^a^

Conditions: 15 min extraction time, 1/20 S/L (g/mL) ratio. U-40: ultrasound at 40 kHz and 300 W. Data are expressed as mean ± standard deviation (*n* = 3). Values with different superscript letters in the same column for the same type of pitaya were found to be significantly different (*p* < 0.05) using Duncan’s multiple range test.

**Table 3:** Effect of solid-to-liquid ratio on the extract yield, total phenolic content (TPC), and ferric-reducing antioxidant power (FRAP) of the dried whole-fruit powders (DPs) obtained from white-flesh (WFP) or red-flesh (RFP) pitaya using U-40.

Type of Pitaya	Solid-to-Liquid Ratio (g/mL)	Extract Yield (% of DP)	TPC (mg GAE/g DP)	FRAP (mg FSE/g DP)
WFP	1/10	67.320 ± 0.864 ^B^	2.125 ± 0.123 ^B^	5.667 ± 0.265 ^A^
	1/20	67.103 ± 1.057 ^B^	2.280 ± 0.111 ^AB^	6.470 ± 0.179 ^A^
	1/30	68.570 ± 1.488 ^B^	2.507 ± 0.190 ^A^	6.078 ± 0.641 ^A^
	1/40	73.360 ± 0.472 ^A^	2.479 ± 0.109 ^A^	6.064 ± 1.222 ^A^
RFP	1/10	52.863 ± 2.125 ^c^	4.552 ± 0.052 ^b^	15.287 ± 0.707 ^b^
	1/20	61.963 ± 1.240 ^ab^	5.568 ± 0.205 ^a^	18.525 ± 1.017 ^a^
	1/30	64.820 ± 0.442 ^a^	5.617 ± 0.306 ^a^	19.407 ± 1.867 ^a^
	1/40	59.480 ± 3.680 ^b^	4.820 ± 0.453 ^b^	17.071 ± 1.861 ^ab^

Conditions: 40 °C, 15 min extraction time. U-40: ultrasound at 40 kHz and 300 W. Data are expressed as mean ± standard deviation (*n* = 3). Values with different superscript letters in the same column for the same pitaya were found to be significantly different (*p* < 0.05) using Duncan’s multiple range test.

**Table 4:** Effect of extraction time on ferric-reducing antioxidant power (FRAP) of the dried whole-fruit powders (DPs) obtained from white-flesh (WFP) or red-flesh (RFP) pitaya for different extraction modes.

Type of Pitaya	Extraction Mode	FRAP (mg FSE/g DP) and Extraction Time			
		5 min	10 min	15 min	60 min
WFP	U-40	6.879 ± 1.086 ^a A^	7.291 ± 1.762 ^a A^	6.470 ± 0.179 ^a A^	5.443 ± 0.192 ^a A^
	U-120	6.745 ± 0.404 ^a A^	6.023 ± 1.090 ^ab A^	5.222 ± 0.184 ^b B^	5.056 ± 0.157 ^b A^
	S-120	6.457 ± 1.645 ^a A^	5.837 ± 1.085 ^a A^	5.560 ± 0.360 ^a B^	5.343 ± 0.591 ^a A^
RFP	U-40	18.834 ± 1.468 ^a A^	17.764 ± 3.166 ^a A^	18.525 ± 1.017 ^a A^	17.443 ± 1.406 ^a A^
	U-120	18.483 ± 1.442 ^a A^	17.750 ± 0.575 ^a A^	17.949 ± 0.329 ^a A^	17.385 ± 1.371 ^a A^
	S-120	18.642 ± 1.355 ^a A^	19.213 ± 1.003 ^a A^	18.256 ± 1.463 ^a A^	17.897 ± 0.248 ^a A^

Conditions: 40 °C, 1/20 S/L ratio (g/mL). U-40: ultrasound at 40 kHz and 300 W; U-120: ultrasound at 120 kHz and 300 W; S-120: shaking at 120 rpm. Data are expressed as mean ± standard deviation (*n* = 3). For the same type of pitaya, values with different uppercase letters for the same extraction time or values with different lowercase letters for the same extraction mode were found to be significantly different (*p* < 0.05) using Duncan’s multiple range test.

**Table 5:** Effect of extraction time on the free chlorogenic acid (CGA) content in the dried whole-fruit powders (DPs) obtained from white-flesh (WFP) or red-flesh (RFP) pitaya for different extraction modes.

Type of Pitaya	Extraction Mode	Free CGA (μg/g DP) and Extraction Time			
		5 min	10 min	15 min	60 min
WFP	U-40	67.78 ± 4.11 ^b B^	75.92 ± 7.53 ^ab A^	101.68 ± 31.12 ^a A^	71.58 ± 5.93 ^ab A^
	U-120	72.00 ± 3.56 ^a AB^	74.13 ± 8.28 ^a A^	76.15 ± 5.89 ^a A^	71.44 ± 5.67 ^a A^
	S-120	75.25 ± 1.40 ^a A^	79.55 ± 2.10 ^a A^	67.85 ± 4.18 ^b A^	65.25 ± 3.90 ^b A^
RFP	U-40	8.68 ± 0.96 ^b A^	11.07 ± 1.08 ^a A^	10.25 ± 1.71 ^ab A^	10.81 ± 0.42 ^ab B^
	U-120	(n.d.) ^c B^	10.82 ± 0.87 ^ab A^	9.63 ± 2.34 ^b A^	12.87 ± 0.18 ^a A^
	S-120	(n.d.) ^c B^	11.05 ± 0.25 ^a A^	8.93 ± 0.82 ^b A^	9.36 ± 0.50 ^b C^

Conditions: 40 °C, 1/20 S/L ratio (g/mL). U-40: ultrasound at 40 kHz and 300 W; U-120: ultrasound at 120 kHz and 300 W; S-120: shaking at 120 rpm. Data are expressed as mean ± standard deviation (*n* = 3). (n.d.): not detected. Values with different uppercase letters for the same extraction time and type of pitaya or values with different lowercase letters for the same extraction mode and type of pitaya were found to be significantly different (*p* < 0.05) using Duncan’s multiple range test.

**Table 6:** Effect of extraction time on the free quercetin content in the dried whole-fruit powders (DPs) obtained from white-flesh (WFP) or red-flesh (RFP) pitaya using different extraction modes.

Type of Pitaya	Extraction Mode	Free Quercetin (μg/g DP) and Extraction Time			
		5 min	10 min	15 min	60 min
WFP	U-40	4.92 ± 0.43 ^b A^	5.51 ± 0.53 ^ab AB^	8.69 ± 3.40 ^a A^	7.17 ± 0.27 ^ab AB^
	U-120	5.55 ± 0.58 ^b A^	4.84 ± 0.90 ^b B^	5.82 ± 0.68 ^b A^	7.67 ± 0.45 ^a A^
	S-120	4.93 ± 0.45 ^c A^	6.24 ± 0.13 ^a A^	5.58 ± 0.44 ^b A^	6.79 ± 0.21 ^a B^
RFP	U-40	(n.d.) ^c^	(n.d.) ^c^	2.26 ± 0.57 ^b A^	3.25 ± 0.37 ^a A^
	U-120	(n.d.) ^c^	(n.d.) ^c^	2.41 ± 0.33 ^b A^	3.00 ± 0.40 ^a AB^
	S-120	(n.d.) ^b^	(n.d.) ^b^	(n.d.) ^b B^	2.42 ± 0.40 ^a B^

Conditions: 40 °C, 1/20 solid-to-liquid ratio (g/mL). U-40: ultrasound at 40 kHz and 300 W; U-120: ultrasound at 120 kHz and 300 W; S-120: shaking at 120 rpm. Data are expressed as mean ± standard deviation (*n* = 3). (n.d.): not detected. Values with different uppercase letters for the same extraction time and type of pitaya or values with different lowercase letters for the same extraction mode and type of pitaya were found to be significantly different (*p* < 0.05) using Duncan’s multiple range test.

## Data Availability

Data are contained within the article.
